# Social-emotional problems in 3-to 5-year-old children: a cross-sectional study of highly educated migrants in a Chinese urban area

**DOI:** 10.3389/fpubh.2024.1357784

**Published:** 2024-07-03

**Authors:** Qingning Xu, Shaoying Liu, Zhijun Zhu, Jingwen Xu, Yujuan Shen, Hongyan Liu, Yanqing Zhou, Luxin Xu

**Affiliations:** ^1^Department of Psychology, School of Science, Zhejiang Sci-Tech University, Hangzhou, China; ^2^Centre for Mental Health Education, Keyi College of Zhejiang Sci-Tech University, Shaoxing, China; ^3^Qiantang District Health Bureau, Hangzhou, China; ^4^Hangzhou Qiantang Xinghua kindergarten, Hangzhou, China; ^5^Hangzhou Qiantang Lingyun kindergarten, Hangzhou, China

**Keywords:** mental health, social-emotional problems, screening, preschoolers, highly educated migrants

## Abstract

**Background:**

Previous research has primarily examined the mental well-being of children from labor migrant families, yet there is a lack of understanding regarding the mental well-being of children from highly educated migrant backgrounds. This study investigated the social-emotional problems of 3-5-year-olds from highly educated migrant families residing in an urban area of China, as well as explored potential differences in demographic variables.

**Methods:**

A cross-sectional study was conducted in Qiantang District, Hangzhou, China, with 1,494 (53.3% boys) children selected via a convenient sampling method. The Ages & Stages Questionnaires: social-Emotional, Second Edition (ASQ:SE-2) was used to measure social-emotional problems.

**Results:**

The results showed that 23.6% of the children were at risk for social-emotional problems. More boys (26.7%) than girls (20.1%) had scores above the cut-off. Additionally, more children in the low socioeconomic status (29.9%) had scores above the cut-off than those in the high socioeconomic status (18.9%). There were three common issues among all age groups: “being more active than others,” “excessive attachment to parents,” and “being overly friendly with strangers.

**Conclusion:**

The social-emotional development of children from highly educated migrant families is a significant aspect that deserves recognition, contributing valuable insights to the existing literature on this topic.

## 1 Introduction

The rapid urbanization in China has led to a notable increase in migrants who are moving to urban areas to seek job opportunities (1–3). China has recently undergone economic restructuring and industrial upgrading (4), with highly educated individuals playing a crucial role in driving national and regional development (5, 6). Therefore, Chinese local governments have implemented public policies to attract and retain highly educated migrants (7–10). It is worth noting that the significance of personal happiness to the younger generation in China is a key factor in their motivation to migrate (7, 11). In fact, to attract and keep talented individuals, it is crucial to take into account not only their own well-being, but also the well-being of their children. However, previous studies have focused on the children of labor migrants (12, 13), it is necessary to also consider the impact of highly educated migrants’ children on urban social dynamics. Unlike labor migrants, highly educated migrants are typically well-educated, have stable jobs and incomes, and can establish themselves in urban areas by purchasing property and obtaining local residency status (14). Consequently, their children are raised in the city (1, 2, 15), where their health and well-being are influenced by various social, psychological, and physical factors (2, 16).

Although highly educated migrant families may have a higher socio-economic status and possess advantages in terms of social adaptation (17), they still face challenges similar to those of labor migrants, such as the stress of migration, loss of social connections (18, 19), and difficulties in forming attachments and identities in their new environment (20). Additionally, the demands of their professional careers may impact their ability to effectively fulfill their roles as parents (21), potentially leading to increased levels of parenting stress (22). These stressors can contribute to feelings of depression and isolation (23), as well as a higher risk of psychological issues among highly educated migrants (24, 25), ultimately affecting the social-emotional development of their children (26).

Identifying and addressing social-emotional problems in early childhood is essential (27–30). This developmental stage is key for children to learn how to express and regulate emotions, build meaningful relationships, and actively explore their environment (31). The social-emotional problems of children in early childhood show considerable stability and are among the most powerful indicators of a child’s future aggression, delinquency, antisocial behavior, and substance abuse (30). Research by Brown et al. (29) found that 24% of preschoolers in the US were at risk for social-emotional problems, while Bian et al. (32) discovered that 14.8% of children aged 36 to 60 months in China were also at risk. Previous studies have primarily examined the mental well-being of children who are left behind by parents working as labor migrants in different locations. A study conducted in impoverished regions of rural China revealed that 33.5% of children aged 0–5 with absent mothers were at risk for social-emotional problems (33), and 30.8% of those under 3 with fathers or parents employed away from home were also prone to such issues (34). However, there is limited research on the mental health of children of highly educated migrants. Neglecting to address social-emotional problems in these children could have long-term consequences (35). Therefore, conducting social-emotional screening for children among highly educated migrants in new urban areas is imperative.

Some commonly used social-emotional screening tools for young children include the Child Behavior Checklist, the Strengths and Difficulties Questionnaire, and The Ages & Stages Questionnaires: Social–Emotional, Second Edition. The ASQ: SE-2 is often preferred for its moderate number of questions, high level of social acceptance, and convenience for large-scale testing (31). However, these screening tools primarily focus on identifying children who may be at risk for social-emotional problems (36, 37). This approach can be problematic as it could potentially lead to stigmatization and parental anxiety for those identified as at risk (38, 39) and may overlook social-emotional concerns in typically developing children. Parents commonly encounter challenging behaviors from their children, such as temper tantrums and clinginess to parents, which may seem minor but can evolve into significant sources of stress (40). As a result, these pressures can have a detrimental impact on children’s development by reducing parental self-efficacy and satisfaction (41). Hence, it is essential to not only screen for children at risk, but also to assess common social-emotional challenges in all children of highly educated migrant parents in order to provide guidance for promoting social-emotional development for all children.

The present study, conducted during the COVID-19 pandemic in 2020–2021, sheds light on the difficulties faced by migrant children. While these children may only experience mild physical symptoms of COVID-19, the disruption to their daily routines has been significant. This includes prolonged school closures, limited access to extracurricular activities and support systems, isolation from friends, and heightened stress and violence within highly educated migrant families (42). Recognizing the mental health needs of these children is essential in providing the necessary support post the COVID-19 pandemic.

This study aimed to investigate the social-emotional problems and identify common social-emotional challenges in 3-to 5-year-old children whose parents are highly educated migrants in Chinese new urban areas. It is well-established that social-emotional problems are linked to social and demographic factors such as gender (43), birth order (44, 45), and socioeconomic status (46). It is crucial to conduct subgroup analyses in order to understand and interpret the social-emotional development and common challenges among children.

## 2 Methods

### 2.1 Design and participants

Data was obtained through a cross-sectional study using convenient sampling from six public kindergarten schools in the Qiantang District of Hangzhou, China from 2020 to 2021. These kindergarten schools were established after the district was formed, and their admission criteria are based on the principle of consistent residence and Hukou registration. Initially, there were 1,815 children enrolled in the six kindergarten schools. Ultimately, a total of 1,494 data points were included in the study. These individuals were the children of highly educated migrants who held college degrees, had moved to urban areas for employment opportunities, and had purchased property. Conversely, 321 data were excluded as their parents were not highly educated migrants.

The Ethics Committee of the Psychology Department at Zhejiang Sci-Tech University has granted permission for the study.

### 2.2 Social-emotional problems

The Ages & Stages Questionnaires: Social-Emotional, Second Edition (ASQ:SE-2) is a parent-reported instrument designed to assess social-emotional problems in children aged 3–66 months (47). ASQ:SE-2 has nine different forms with items suited to a child’s age. These questionnaires cover various psychological domains, including self-regulation, compliance, adaptive functioning, autonomy, affect, social communication, and interpersonal interaction. Using a three-point Likert scale (10 = most of the time, 5 = sometimes, 0 = never or rarely) to rate each item, an additional score of 5 is added when the item is concerning for parents. If the total score is equal to or higher than the cut-off point, the child is considered to be “at risk for social-emotional problems,” meaning they need further evaluation and/or intervention. Items with more than 10% of the highest scores (10 or 15 points) are identified as common social-emotional challenges in children. Depending on the age of the child, we used three different questionnaires: one for 3-year-olds (33 months 0 days to 41 months 30 days; 31 items), another for 4-year-olds (42 months 0 days to 53 months 30 days; 33 items), and a third for 5-year-olds (54 months 0 days to 71 months 30 days; 33 items). The internal consistency coefficients for each age group in the current study were 0.95, 0.96, and 0.95, respectively.

### 2.3 Sociodemographic

The sociodemographic data gathered included age, gender, birth order, parents’ occupations, and educational levels. The parents’ occupations were assigned 1–5 points according to the criteria for the relevant occupation classification. The educational levels of parents were given 1–5 points according to “junior high school or below,” “senior high school or technical secondary school,” “junior college,” “undergraduate,” and “graduate” or above. This study also examined the effects of family socioeconomic status on children’s social-emotional problems (48). Socioeconomic status was measured by the scores of the parents’ occupations and education levels, ranging from 4 to 20. The average score ± 1 standard deviation of socioeconomic status was used to distinguish different levels of socioeconomic status. One standard deviation higher than the average score was considered high socioeconomic status, and one standard deviation lower than the average score was considered low socioeconomic status; the rest were considered middle socioeconomic status.

### 2.4 Data analyses

We used descriptive and comparative statistical analyses. Descriptive results were presented as numbers (n), means (M), standard deviations (SD), ranges, and percentages (%). We calculated the total scores and used the cut-off values to detect children with social-emotional problems according to the instructions in the ASQ: SE-2 User’s Guide (47). Logistic regression models were utilized to study the associations between participant characteristics and social-emotional problems in children. The significance level chosen for this study was 0.05. The data were analyzed using SPSS Statistics version 22.0.

## 3 Results

Table 1 displays the detailed demographic characteristics of the participants. The majority of the questionnaires were completed by mothers (86.9%), while the remaining were filled out by fathers (13.1%). The distribution of children in the study was as follows: 15.2% were 3-year-old group, 35.3% were 4-year-old group, and 49.5% were 5-year-old group, with 53.3% being boys. Furthermore, 73.9% of the children were first-borns. In terms of parental occupation, 87.0% of fathers and 72.4% of mothers were classified as clerical workers/low-ranking officials or above. Additionally, 91.7% of fathers and 89.1% of mothers had an education level of associate college or higher. The study also found that 61.7% of families belonged to middle-socioeconomic-status, while 19.5% were classified as high-socioeconomic-status.

**Table 1:** Participant characteristics (*n* = 1,475).

Characteristics		*n*	%
Age	3-year-old	227	15.2
	4-year-old	527	35.3
	5-year-old	74	49.5
Gender	Boys	797	53.3
	Girls	697	46.7
Birth order	1	1,104	73.9
	≥2	390	26.1
Fathers’ occupation	Lay-offs or the unemployed	8	0.5
	Physical laborers	186	12.4
	Low-ranking officials	345	23.1
	Middle-ranking officials	613	41.0
	High-ranking officials	342	22.9
Fathers’ education	Junior middle school and below	24	1.6
	Senior high school	101	6.8
	Associate College	349	23.4
	Undergraduate	828	55.4
	Postgraduate and above	192	12.9
Mothers’ occupation	Lay-offs or the unemployed	298	19.9
	Physical laborers	115	7.7
	Low-ranking officials	488	32.7
	Middle-ranking officials	500	33.5
	High-ranking officials	93	6.2
Mothers’ occupation	Junior middle school and below	26	1.7
	Senior high school	142	9.5
	Associate College	445	29.8
	Undergraduate	719	48.1
	Postgraduate and above	162	10.8
Family socioeconomic status	Low	281	18.8
	Middle	922	61.7
	High	291	19.5

A total of 23.6% of children scored above the recommended cut-off on the ASQ: SE. The mean scores for the 3-, 4-, and 5-year-old groups were 58.50 ± 28.80, 49.31 ± 27.95, and 55.12 ± 35.73, respectively, with scores ranging from 10 to 185, 0 to 165, and 0 to 255. In each age group, a different percentage of children scored above the cut-off: 34.4, 14.8, and 26.6%. More boys than girls had scores above the cut-off (26.7% compared to 20.1%) (OR = 1.49; 95% CI, 1.17–1.90; *p* < 0.001). Additionally, more children in the low-socioeconomic-status (29.9%) (OR = 1.50; 95%CI, 1.10 ~ 2.04; *p* = 0.01) had scores above the cut-off than those in the high-socioeconomic-status (18.9%) (Table 2).

**Table 2:** Associations of the proportion of children at risk for ASQ:SE and participant characteristics (*n* = 1,475).

Characteristics	Below the cut-off (%)	Above the cut-off (%)	OR (95%CI)	*p*
Gender				
Girls	557 (79.9)	140 (20.1)	1.00	
Boys	584 (73.3)	213 (26.7)	1.49 (1.17 ~ 1.90)	<0.001
Birth order				
1	841 (76.2)	263 (23.8)	1.00	
≥2	300 (76.9)	90 (23.1)	0.83 (0.63 ~ 1.11)	0.214
Socioeconomic status				
High	236 (81.1)	55 (18.9)	1.00	
Middle	708 (76.8)	214 (23.2)	0.75 (0.54 ~ 1.05)	0.09
Low	197 (70.1)	84 (29.9)	1.50 (1.10 ~ 2.04)	0.01

Parent-reported challenges were identified across all age groups, with three common items emerging. In the self-regulation domain, the question “Does your child seem more active than other children?” was a challenge for 29.5% of children in the 3-year-old group, 27.5% in the 4-year-old group, and 27.2% in the 5-year-old group. In the autonomy domain, the question “Does your child cling to you more than you expect?” was a challenge for 27.3, 23.0, and 24.6% of children in each age group, respectively. In the interpersonal interaction domain, the question “Does your child seem too friendly with strangers?” was a challenge for 14.5, 12.7, and 17.8% of children in each age group, respectively. Additional social-emotional challenges were identified for the 3-year-old group, including difficulties transferring between activities, eating problems, and unpleasantness in mealtimes, with proportions ranging from 11.0 to 12.8%. No additional challenges were reported for the 4-year-old group. In the 5-year-old group, an additional challenge related to eating problems was reported by 10.9% of parents.

The common social-emotional challenges stratified by gender, birth order, and socioeconomic status are shown in Table 3. Boys (32.1%) were found to be more active than girls (22.5%) (OR = 1.67; 95% CI, 1.32–2.10; *p* < 0.001). Non-firstborns (23.6%) are not significantly more active than firstborns (29.1%) (OR = 0.68; 95% CI, 0.52–0.90; *p* = 0.007), while non-firstborns were found to be more attached to their parents than firstborns (29.50% vs. 22.60%; OR = 1.35; 95% CI, 1.03–1.77; *p* = 0.029). Additionally, children in the low socioeconomic status (31.0%) were more active than those in the high socioeconomic status (27.5%) (OR = 1.38; 95% CI, 1.02–1.87; *p* = 0.037). Meanwhile, children in the middle socioeconomic status (18.6%) (OR = 1.46; 95% CI, 1.03–2.10; *p* = 0.034) and low socioeconomic status (19.6%) (OR = 1.64; 95% CI, 1.15–2.36; *p* = 0.007) were more overly friendly with strangers than those in the high socioeconomic status (13.3%).

**Table 3:** Associations of the proportion of children at risk for social-emotional problems and participant characteristics (*n* = 1,475).

Characteristics	Does your child seem more active than other children?		Does your child cling to you more than you expect?		Does your child seem too friendly with strangers?	
	OR (95%CI)	*p*	OR (95%CI)	*p*	OR (95%CI)	*p*
Gender						
Girls	1.00		1.00		1.00	
Boys	1.67 (1.32 ~ 2.10)	<0.001	0.84 (0.66 ~ 1.07)	0.154	1.05 (0.79 ~ 1.40)	0.720
Birth order						
1	1.00		1.00		1.00	
≥2	0.68 (0.52 ~ 0.90)	0.007	1.35 (1.03 ~ 1.77)	0.029	0.87 (0.62 ~ 1.21)	0.402
Socioeconomic status						
High	1.00		1.00		1.00	
Middle	1.00 (0.74 ~ 1.37)	0.992	0.85 (0.61 ~ 1.17)	0.310	1.46 (1.03 ~ 2.10)	0.034
Low	1.38 (1.02 ~ 1.87)	0.037	1.25 (0.92 ~ 1.70)	0.158	1.64 (1.15 ~ 2.36)	0.007

## 4 Discussion

This study examined the mental health of young children aged 3–5 years old whose parents were highly educated migrants living in a new urban area in China. According to parental reports, 23.6% of the children were identified as being at risk for social-emotional problems, with boys more likely to be at risk than girls. Children from low socioeconomic status were also found to be at higher risk compared to those from higher socioeconomic status. The most commonly reported challenges included hyperactivity, clinginess, and friendliness toward strangers. Boys were more likely to display hyperactive behavior, non-firstborn children were more likely to be clingy, and children from both low and high socioeconomic status were more likely to be friendly toward strangers compared to those from middle socioeconomic status.

This study is the first to focus on the social-emotional problems of children of highly educated migrants in urban areas. The results showed that 23.6% of 3-to 5-year-olds were at risk, which is lower than the 33.1% of children in rural areas of central and western China (33). However, it is higher than the norm for children aged 2–5 in China (14.8%) (32), but similar to the reported prevalence among US preschoolers (24%) (29). The lower incidence of social-emotional problems among the offspring of highly educated migrants compared to Chinese labor migrants is likely due to differences in family socioeconomic status. Previous research has shown a correlation between children’s social-emotional problems and family socioeconomic status (17, 48). The higher incidence of social-emotional problems among the children of highly educated migrants compared to the Chinese norm may be attributed to the pressures of immigration and changes in social relations (49), which can challenge children’s emotional socialization (18, 19).

This study found three common social-emotional issues among 3-5-year-old children of urban highly educated migrants in China: (1) being more active than others; (2) being overly attached to their parents; and (3) being too friendly with strangers. Previous studies in Sweden have also revealed that 3-year-olds commonly display behaviors such as being more active than others, expressing strong emotions, and returning to their parents in new situations (45). These social-emotional issues are not limited to children at risk, but are common challenges for all children. These concerns are particularly relevant for migrant parents, as they can lead to minor parenting stress and impact parent–child interactions, ultimately affecting the child’s social-emotional development. Both Bronfenbrenner’s ecological theory (50) and Denham’s emotional socialization model (51) highlight the importance of parent–child interactions in a child’s development. Highly educated migrant parents who are focused on work pursuits (52) may struggle to find time for family activities (53, 54) leading to these social-emotional problem behaviors. Without the related knowledge and strategies to address these issues, there is a potential risk of social-emotional difficulties accumulating over time (45).

While highly educated migrant children in the study had a lower risk incidence compared to labor migrant children, nearly a quarter of all children were still at risk for social-emotional problems. This is concerning because social-emotional issues in early childhood can have long-lasting effects on mental health in adolescence. Additionally, three common social-emotional challenges are not exclusive to children at risk, but are prevalent among all children, indicating that over 10% of parents experience difficulties in managing their children’s behaviors. The ASQ:SE-2 demonstrates strong psychometric properties (16), with each item evaluating children’s typical social-emotional functioning and identifying potential signs of future issues or disabilities. According to Crnic’s research (40), while these challenges may seem minor, they can gradually diminish parenting efficacy and satisfaction. This, in turn, could impact the quality of parent–child interactions and potentially hinder children’s social and emotional development. Hence, it may be beneficial to conduct a more comprehensive evaluation of children facing these challenges in the future.

Given the current circumstances, it appears that the trend of raising children of highly educated migrants will persist in new urban areas for the foreseeable future. This study sought to expand our understanding of the social-emotional development of children from highly educated migrant families in new urban areas, a group often overlooked in research. However, there are some limitations to consider. Firstly, the study utilized a convenience sampling method to explore the social-emotional issues of 3-5-year-olds from highly educated migrant families in an urban area of China, without comparing them to local children in the same area. Additionally, the study relied solely on parental reports, which could introduce response bias. To obtain a more comprehensive understanding of children’s social-emotional challenges, future research should include a more diverse and representative sample, as well as incorporate multiple methods of data collection, such as independent observer observations and teacher evaluations.

## 5 Conclusion

In summary, this study examined the social-emotional problems encountered by children from highly educated migrant families in a new urban area in China. It identified the social-emotional problems and common challenges that exist within different groups of these children. The findings contribute to broadening research on the social-emotional development of highly educated migrant children and emphasize the importance of being aware of the mental health of these children.

## Data availability statement

The raw data supporting the conclusions of this article will be made available by the authors, without undue reservation.

## Ethics statement

The studies involving human participants were reviewed and approved by the Psychology Ethics Committee of Zhejiang Sci-Tech University. The participants’ legal guardian/next of kin provided their written informed consent to participate in this study.

## Author contributions

QX: Writing – original draft, Methodology, Investigation, Formal analysis, Data curation, Conceptualization. SL: Writing – review & editing, Supervision, Resources, Funding acquisition, Formal analysis, Conceptualization. ZZ: Writing – review & editing, Supervision, Methodology, Conceptualization. JX: Writing – original draft, Supervision, Methodology, Data curation. YS: Writing – original draft, Supervision, Methodology, Data curation. HL: Writing – review & editing, Supervision, Investigation, Conceptualization. YZ: Writing – review & editing, Resources, Investigation, Data curation. LX: Writing – review & editing, Supervision, Resources, Investigation.
